# Supplementary material for: Calibrated rare variant genetic risk scores for complex disease prediction using large exome sequence repositories
Source: Nat Commun. 2021 Oct 6;12:5852. doi: 10.1038/s41467-021-26114-0 (PMC8494733; doi:10.1038/s41467-021-26114-0)
Supplement: Supplementary file 1 — Supplementary Information [file 41467_2021_26114_MOESM1_ESM.pdf]

Supplementary Information

Calibrated rare variant genetic risk scores for complex disease prediction using large exome sequence repositories

Ricky Lali [1,2], Michael Chong [1,3], Arghavan Omidi [1], Pedrum Mohammadi-Shemirani [1,4], Ann Le [1,4], Edward Cui [1], and Guillaume Paré [1-7]

1 Population Health Research Institute, David Braley Cardiac, Vascular and Stroke Research Institute, 237 Barton Street East, Hamilton, ON L8L 2X2, Canada
2 Department of Health Research Methodology, Evidence, and Impact, McMaster University, Faculty of Health Sciences, 1280 Main Street West, Hamilton ON L8S 4K1, Canada
3 Department of Biochemistry and Biomedical Sciences, McMaster University, Faculty of Health Sciences, 1280 Main Street West, Hamilton ON L8S 4K1, Canada
4 Department of Medical Sciences, McMaster University, Faculty of Health Sciences, 1280 Main Street West, Hamilton ON L8S 4K1, Canada
5 Thrombosis and Atherosclerosis Research Institute, David Braley Cardiac, Vascular and Stroke Research Institute, 237 Barton Street East, Hamilton, ON L8L 2X2, Canada
6 Department of Pathology and Molecular Medicine, McMaster University, Michael G. DeGroote School of Medicine, 1280 Main Street West, Hamilton ON L8S 4K1, Canada
7 Department of Clinical Epidemiology & Biostatistics, McMaster University, 1280 Main Street West, Hamilton ON L8S 4K1, Canada

Supplemental Tables: 9
Supplemental Figures: 15
Supplemental References: 16

Corresponding Author:
Guillaume Paré MD, MSc, FRCPC
McMaster University
Population Health Research Institute
David Braley Cardiac, Vascular, and Stroke Research Institute
237 Barton Street East – C4 126
Phone: 905-527-4322 x40365; Fax 905-297-3789
E-mail: pareg@mcmaster.ca

Contents

Supplementary Note 1: Data acquisition and discovery study samples………………….. **1**
Supplementary Note 2: Broad exome sequencing……………………………………….. **4**
Supplementary Note 3: Variant-level QC for MIGen and gnomAD……………………... **5**
Supplementary Note 4: Variant-level QC for only MIGen……………………………….. **6**
Supplementary Note 5: Sample-level QC……………………………………………… **7**
Supplementary Note 6: Validation study populations……………………………… **9**
Supplementary Note 7: Data processing……………………………………………….. **11**
Supplementary Figures……..………………………………………………………….. **14**
Supplementary Figure 1: Simulated effects of SFN rate, SFP rate, and PSF on estimated OR and association power…………………………………………………………….. **15**
Supplementary Figure 2: Simulated effect of combined SFN rate, SFP rate, and high PSF on estimated OR……………………………………………………………………….. **16**
Supplementary Figure 3: Power to detect gene-based association signals in the presence of SFN rate, SFP rate, and PSF…………………………………………………………… **17**
Supplementary Figure 4: Number of variant sites remaining in (a) gnomAD, (b) GIAB, and (c) MIGen after intersection with different region intervals…………………………… **18**
Supplementary Figure 5: GIAB iCF values for gene groups stratified by decile of gene constraint score……………………………………………………………………………... **19**
Supplementary Figure 6: iCF values for cases and controls across discovery MIGen cohorts………………………………………………………………………………………… **20**
Supplementary Figure 7: gCF values across 50 gene bins derived from 3,352 healthy MIGen controls……………………………………………………………………………... **21**
Supplementary Figure 8: Calibration of gene-based test statistics using quality-by-depth (QD) scores for rare synonymous SNVs according to the TRAPD algorithm for 3,352 healthy control participants in MIGen……………………………………………………… **22**
Supplementary Figure 9: Dispersion of delta counts generated using RV-EXCALIBER or TRAPD across the spectrum of EAC for 3,352 healthy control participants in MIGen… **23**
Supplementary Figure 10: Quantile-quantile plots for gene-based test statistics generated using RV-EXCALIBER base and a Fisher's Exact test for 3,352 healthy control participants in MIGen ……………………………………………………………………… **24**
Supplementary Figure 11: Quantile-quantile plots for gene-based test statistics generated using RV-EXCALIBER base, RV-EXCALIBER iCF and RV-EXCALIBER iCF and gCF (i.e. fully adjusted model) for 3,352 healthy control participants in MIGen… **25**
Supplementary Figure 12: Predictive effect of TRAPD and RV-EXCALIBER-derived RVGRS on simulated case status in the UK Biobank……………………………………… **26**
Supplementary Figure 13: Workflow for benchmark simulations to assess the predictive power of the RV-EXCALIBER and TRAPD frameworks ………………………………… **28**
Supplementary Figure 14: Distribution of RVGRS950^{LDLR-} among CAD-free controls, general CAD cases, and early CAD cases in the UK Biobank…………………………….. **29**
Supplementary Figure 15: Predictive effect and prevalence of CAD across increasing RVGRS950 and RVGRS950^{LDLR-} percentile groupings in the UK Biobank ……………… **30**
Supplementary Tables………………………………………………………………….. **31**
Supplementary Table 1: Baseline and sequencing characteristics for individual cohorts used from the MIGen consortium……………………………………………………….... **32**

Supplementary Table 2: Ethnicities in gnomAD used to generate the EAC for a given GIAB sample………………..……………………………………………………………... **33**
Supplementary Table 3: iCF values for every GIAB vs. gnomAD ethnicity across 4 allele frequency bins. ………………………………………………………………………. **34**
Supplementary Table 4: Median iCF values for healthy control participants across 8 MIGen cohorts. ...……………………………………………………………………………... **35**
Supplementary Table 5: Predictive effect estimates of iCF distribution in MIGen control versus case participants.……………………... **36**
Supplementary Table 6: Gene-set enrichment for nominally associated (P<0.05) discovery genes from RV-EXCALIBER…………………………………………….......... **37**
Supplementary Table 7: Predictive effect of RVGRS950 and RVGRS950^{LDLR-} on CAD across tertiles of FRS in the UK Biobank..………………………………………………… **38**
Supplementary Table 8: Predictive effect of CVGRS on tertiles on CAD across tertile of FRS in the UK Biobank………………………………………………………………….. **39**
Supplementary Table 9: Net reclassification improvement index when incorporating RVGRS950 and RVGRS950^{LDLR-} to risk models………………………………………... **40**
Supplementary references…………………………………………………………... **41**

Supplementary Note 1: Data acquisition and discovery study samples

A. genome Aggregation Database (gnomAD)

The gnomAD dataset consists of 120,393 exomes which were aggregated across 35 exome sequencing consortia[1]. Release 2.0.1 of the gnomAD dataset (gnomAD r2.0.1) was used as the comparator population against all individual-level sequences (described in Supplementary Note 1, sections B-C) and was obtained in variant call file format from Google Cloud public datasets (https://gnomad.broadinstitute.org/downloads). After variant-level quality control (QC) (discussed in Supplemental Note 3, sections A-C), there were a total of 13.1 million variant calls (93.6% single nucleotide variants (SNVs); 6.4% insertions/deletions (INDELs)).

B. Genome In A Bottle (GIAB) consensus sequences

The GIAB consortium is hosted by the National Institute of Standards and Technology (NIST) to provide gold-standard reference samples for benchmarking human genomic sequences[2]. To evaluate the role of population-specific effects to exome-wide total allele count, we determined correction factors using high-confidence sequence variants corresponding to 3 reference GIAB samples of different ethnicities (using methodology developed by Zook *et al.* 2019)[3] : 1) NA12878 (North-western European; NIST ID HG001 [https://ftp-trace.ncbi.nlm.nih.gov/giab/ftp/release/NA12878_HG001/NISTv3.3/]), 2) NA24631 (East Asian; NIST ID HG005 [https://ftp-trace.ncbi.nlm.nih.gov/giab/ftp/release/ChineseTrio/HG005_NA24631_son/NISTv3.3/]), and 3) NA24385 (Ashkenazi Jewish; NIST ID HG002 [https://ftp-trace.ncbi.nlm.nih.gov/giab/ftp/release/AshkenazimTrio/HG002_NA24385_son/NISTv3.3.2/GR

Ch37/]). Variants called in these reference samples were harmonized across 5 sequencing technologies (Illumina, Complete Genomics, Ion Torrent, SOLiD, and 10X Genomics), which collectively use 5 read mappers and 4 variant callers to generate a "consensus" variant callset that we used as benchmark sequences to evaluate the excess or deficit in total allele count across all protein-coding genes in the gnomAD database. Consensus variant calls also allows for the identification of "difficult-to-sequence" regions across the genome, such as segmental duplications, short tandem repeats and other structural variations. Exclusion of these regions can subsequently be used to generate "high-confidence" genomic intervals. High confidence region files and variant call sets (in variant call format) for version 3.3 of NA12878 [https://ftp-trace.ncbi.nlm.nih.gov/giab/ftp/release/ChineseTrio/HG005_NA24631_son/NISTv3.3/], version 3.3 of NA24631 [https://ftp-trace.ncbi.nlm.nih.gov/giab/ftp/release/ChineseTrio/HG005_NA24631_son/NISTv3.3/], and version 3.3.2 of NA24385 [https://ftp-trace.ncbi.nlm.nih.gov/giab/ftp/release/AshkenazimTrio/HG002_NA24385_son/NISTv3.3.2/GRCh37/] were obtained from the GIAB ftp repository for use in our benchmarking analysis.

C. Myocardial Infarction Genetics exome sequencing consortium (MIGen)

MIGen exome sequencing datasets were obtained from the database of Genotypes and Phenotypes (dbGaP). All data handling and analyses were approved by the Hamilton Integrated Research Ethics Board and approval for data downloads was subsequently granted by the National Heart, Lung, and Blood Institute Data Authorization Committee (NHLBI DAC). All participants provided informed consent to the NHLBI DAC for genetic and phenotypic data to be used for health, medical, and biomedical purposes. Variant call files (VCFs) and general phenotypic

information were downloaded with authorized access using version 2.9 of NCBI's Sequence Read Archive (SRA) toolkit[4]. All dbGaP study accessions used in this work are listed in Supplementary Table 1 for reference. A total of 9 MIGen cohorts were downloaded from dbGaP. Summary information on each cohort (including dbGaP accession codes and hyperlinks) is provided in Supplementary Table 1.

Supplementary Note 2: Broad exome sequencing

All exome sequencing was performed at the Broad Institute of Harvard and MIT. Sample sequence capture chemistry and sequencing platforms are stated for each MIGen cohort in Supplementary Table 1. All methodology pertaining to 1) QC of sample DNA, 2) exome sequencing, 3) library construction and in-solution hybrid selection, 4) preparation of libraries for cluster amplification and sequencing, 5) cluster amplification and sequencing, 6) read mapping and variant analysis, 7) sequencing QC, or 8) variant calling has been extensively described in previous works[5–7].

Supplementary Note 3: Variant-level QC for MIGen and gnomAD

A. Data preparation

All multi-allelic variants were broken into separate records using vcflib's vcfmultibreak tool[8] and were treated as separate variants. All INDEL variants were left-aligned using the bcftools norm function to standardize reference and alternate allele INDEL calls according to the hg19 reference genome[9].

B. Filter sites

Only sites receiving a filter notation of "PASS" were kept in downstream analyses. As such, sites failing any variant quality metric were eliminated.

C. Allele counts

Only variant sites demonstrating at least one heterozygous carrier (i.e. an allele count of ≥ 1 were retained.

Supplementary Note 4: Variant-level QC for only MIGen

A. Hardy-Weinberg Equilibrium

Variants deviating significantly from Hardy-Weinberg equilibrium ($P < 5 \times 10^{-6}$) were removed from the analysis. It is important to note that variants considered for Hardy-Weinberg equilibrium were processed through variant level QC described in Supplementary Note 3, sections A-C.

B. Variant missingness

No variant-level missingness threshold was applied as variants exhibiting high missingness would implicitly be removed using the internal allele-frequency filter as discussed in Supplementary Note 7, section E.

Supplementary Note 5: Sample-level QC

A. Sample missingness

Rate of missing genotype calls were determined for each sample in each MIGen cohort. Since the distribution of missingness can vary between studies due to batch effects, samples exhibiting missingness > 6 standard deviations (SDs) from the mean within each MIGen cohort were removed.

B. Sex check

Sex check was performed in plink version 1.9 [10] on a cohort-by-cohort basis. Method-of-moments F coefficients based on observed and expected homozygosity counts in the X-chromosome were calculated after removing the pseudo-autosomal regions. Reported females demonstrating F coefficients greater than 6 SDs and reported males demonstrating F coefficients less than 6 SDs were removed from the analysis.

C. Ethnicity check

Using the genetic complex trait analysis (GCTA) tool[11], principal component analysis was conducted with common variants (minor allele frequency (MAF) >0.01) passing QC from each study. Principal components were projected onto the backdrop of 1000Genomes phase 3 samples, corresponding to reference clusters of African (n=661), East Asian (n=504), European (n=503), South Asian (n=489), and Latin American (n=347) ancestry. Scatter plots of the first two principal components were inspected to identify samples with discrepant reported vs. genetic ethnicity.

D. Kinship

Kinship analyses were performed using KING[12] in each cohort separately. Kinship analysis was automatically stratified by reported ethnic group since no individual cohort had a mixture of reported ancestries. Variants contributing to the kinship analysis were restricted to those that 1) were autosomal 2) were SNVs 3) were designated 'PASS' sites, 4) had a MAF > 0.01, and 5) had < 10% sample missingness. Variants meeting all of the above criteria were further pruned according to linkage disequilibrium. Specifically, r^2 values were determined in a pairwise fashion for these variants using a window size of 50 and a step size of 5. One variant in a given pair would be removed if the r2 value exceeded 0.2. According to KING documentation, pairs of samples with estimated kinship coefficient ranges of >0.354, [0.177 - 0.354], [0.0884 - 0.177] and [0.0442 - 0.0884] were designated as duplicates/MZ twins, 1st-degree relatives, 2nd-degree relative, and 3rd-degree relatives, respectively. As such, a single sample from a pair with a kinship coefficient ≥0.0442 (that which had less overall missingness) was kept.

E. Heterozygosity

A method-of-moments F-coefficient based on the observed and expected homozygous genotypes was determined using plink v1.9[10] using the same set of variants as used in the kinship analysis. F-coefficients demonstrating values less or greater than 6 SDs from the mean were removed as likely sources of sample admixture and consanguinity, respectively.

Supplementary Note 6: Validation study populations

A. UK Biobank

The prospective UK Biobank whole-exome sequencing data release was accessed in March 2019 under application #15255 and contained individual-level genotype data for all individuals who consented for genetic analysis. All data handling and analyses were approved by the Hamilton Integrated Research Ethics Board and approval. The UK Biobank study received approval from the National Health Service National Research Ethics Service North West. The pVCF plink binary format files generated using the Functional Equivalent pipeline were downloaded using ukbfetch[13]. After performing variant and sample-level QC as previously described in Supplementary Notes 3-5, we achieved 45,850 unrelated subjects of European ancestry (i.e. British and other Caucasians as ascertained by principle component analysis) and 16,051 protein-coding genes with a least singleton variant (as per variant filtering criteria described in Supplementary Note 3, section C). Disease phenotypes in the UK Biobank were defined according to International Classification of Disease, Tenth Revision (ICD-10) (UK Biobank data field 41270). In particular, coronary artery disease (CAD) was evaluated using a liberal composite outcome consisting of angina, acute and chronic ischemic heart disease, myocardial infarction (MI), or coronary revascularization. Angina pectoris, ischemic heart disease, and myocardial infarction was based on either self-report or hospital admission diagnosis. Angina pectoris was ascertained using ICD-10 codes I20.X. Acute and chronic ischemic heart disease was ascertained using ICD-10 codes I24.0, I24.8-24.9 and I25.0-25.1, I25.6, I25.8-25.9, respectively. MI was ascertained using ICD-10 codes of I21.X, I22.X, I23.X, I24.1, I25.2. Coronary revascularization was assessed based on an OPCS-4 coded

procedure for coronary artery bypass grafting (K40.1–40.4, K41.1–41.4, K45.1–45.5) or coronary angioplasty with or without stenting (K49.1–49.2, K49.8–49.9, K50.2, K75.1–75.4, K75.8–75.9). Irritable bowel disease (used as a negative control phenotype) was defined according to ICD-10 codes of K85.0 or K85.9.

B. Pakistani Risk of Myocardial Infarction Study (PROMIS)

PROMIS represents a single a sub-study among the entire MIGen cohort (see Supplementary Note 1, section C), but was selected *a priori* as a validation population. As such, the variant and sample-level QC are identical to what was described above in Supplementary Notes 3-5. The dbGaP accession code and associated hyperlink for the PROMIS cohort can be found in Supplementary Table 1.

Supplementary Note 7: Data processing

A. Variant annotation

All variants underwent gene-based annotation using the ANNOVAR *geneanno* pipeline with the refGene database [14]. Specifically, variants were classified to 1 of 9 genomic regions: 1) exonic, 2) splice donor and acceptor sites, 3) non-coding RNA (ncRNA), 4) untranslated region at the 5-prime end (UTR5), 5) untranslated region at the 3-prime end (UTR3), 6) intronic, 7) upstream, 8) downstream, or 9) intergenic. All variants were annotated to the gene harbouring the variant in question. Exonic variants were further classified into 8 categories: 1) frameshift insertion, 2) frameshift deletion, 3) stopgain, 4) stoploss, 5) nonframeshift insertion, 6) nonframeshift deletion, 7) nonsynonymous SNV, 8) synonymous SNV. Nonsynonymous SNVs were annotated with version 1.0 of the Mendelian Clinically Applicable Pathogenicity (M-CAP) score [15] for pathogenic classification. Lastly, variants were annotated according to their corresponding alternate allele frequencies in version 2.0.1 of gnomAD.

B. Variant pathogenicity filtering for GIAB consensus sequences and gnomAD

Due to the depletion of variants in the GIAB consensus sequences after filtering with the intersection of HCC and NIST high-confidence sites, analysis was restricted to all exonic SNVs and there was no formal pathogenicity criteria applied for variant inclusion. These criteria were applied to variants present in the GIAB consensus sequences and gnomAD.

C. Variant frequency filtering for GIAB consensus sequences and gnomAD

Each variant within the GIAB consensus sequence was annotated with its corresponding allele

frequency in a closely related population within gnomAD. Specifically, the North-western European GIAB sample (NA12878) was annotated with the Non-Finnish European (NFE) population allele frequencies, the Ashkenazi Jewish GIAB sample (NA24385) was annotated with both the NFE and Ashkenazi Jewish (ASJ) population allele frequencies, and the East Asian GIAB sample (NA24631) was annotated with the East Asian (EAS) population allele frequency. Since the gnomAD supplies the alternate allele frequency (AAF) for each variant, we decided to standardize all annotated variants to the minor allele as opposed to eliminating all variants with an AAF > 0.5. After standardization of gnomAD allele frequency annotations, variants from the GIAB consensus sequences and gnomAD were stratified into 4 MAF threshold bins: 1) 0 ≤ MAF ≤ 0.01 (rare variants), 2) 0.01 ≤ MAF ≤ 0.05 (low-frequency variants), 3) 0.05 ≤ MAF ≤ 0.25 (common variants), and 4) 0.25 ≤ MAF ≤ 0.5 (very common variants).

D. Variant pathogenicity filtering for MIGen, UK Biobank, and gnomAD

Percentiles were established for the M-CAP scores of all 71,561,086 nonsynonymous variants in the exome. Variants in were thereafter filtered for nonsynonymous (i.e. missense) SNVs with an M-CAP score of >0.025 (50th percentile; which is also the default score for ascribing a nonsynonymous SNV as pathogenic[15]) plus disruptive variants, which included variants leading to a premature stop codon (stopgain), insertion or deletion variants altering the DNA reading frame (frameshift insertion/deletion) and SNVs within splice donor/acceptor sites (splicing).

E. Variant frequency filtering for MIGen, UK Biobank, and gnomAD

All qualifying variants were annotated to their corresponding AAFs in the 5 major gnomAD ancestries: 1) Non-Finnish European, 2) African, 3) South Asian, 4) East Asian, and 5) Latin

American. Prior to filtering, all AAFs annotated using gnomAD were standardized to the minor allele as stated in Supplementary Note 7, section C. All MIGen and UK Biobank sequence variants were also annotated with cohort-specific AAFs (which were also standardized to the minor allele) in order to mitigate batch effects due to non-biological factors (e.g. technological biases intrinsic to a given sequencing technology or variant caller). All MIGen sequence variants with a MAF < 0.001 in all 5 major gnomAD ancestries and in their specific cohorts were kept for downstream analysis. The same threshold was applied to variants in gnomAD, but these variants were not annotated with MIGen or UK Biobank cohort-specific frequencies.

Supplementary Figures

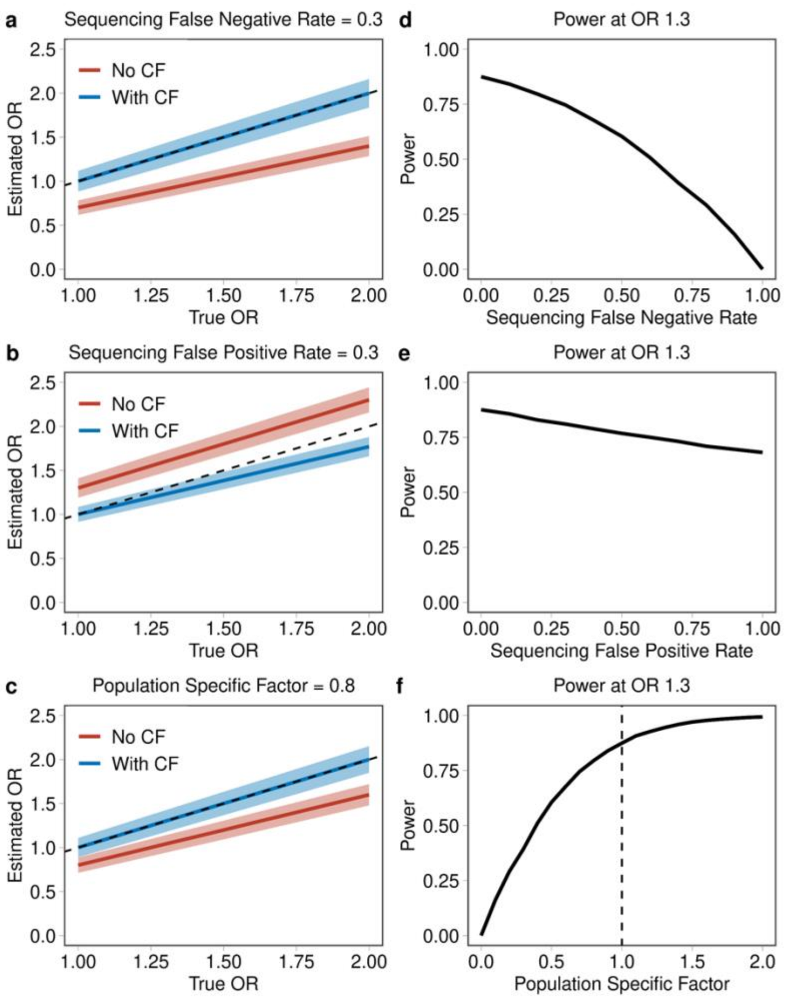

Supplementary Figure 1: Simulated effects of SFN rate, SFP rate, and PSF on estimated OR and association power. Probability of mutation (*P*) within a gene was calculated using equation 2 by simulating true OR values from 1 to 2 in 0.1 stage intervals while keeping SFN, SFP and PSF fixed. Thereafter, a mean estimated OR was calculated for every unique *P* (**a-c; red lines**) across 100,000 simulations**.** CF values were calculated as the ratio of the *P* for each estimated OR to the original cumulative minor allele frequency for a single gene ($CMAF_{Gene}$; set at 0.05) according to equation 3. Each CF was then used to adjust the $CMAF_{Gene}$ to calculate an adjusted *P* (*P**) according to equation 4 while keeping SFN, SFP, and PSF fixed at the same values. Using P^*, a CF-adjusted mean estimated OR was calculated (**a-c; blue lines**) across 100,000 simulations. Dashed lines in **a-c** indicate the line of expectation. Shaded regions in a-c correspond to the 95% confidence interval for the mean estimated OR. CF-adjusted power curves were generated using different ranges of SFN (0 to 1), SFP (0 to 1), and PSF (0 to 2) at a fixed true OR value of 1.3 (**d-f**). Power for every value of SFN, SFP, and PSF was calculated as the proportion of all simulations with a *P*-value < 0.05. Dashed lines in **f** represents a null deviation in PSF. OR indicates odds ratio, CF indicates correction factor, SFN indicates sequencing false negatives, SFP indicates sequencing false positives, PSF indicates population-specific factor. Equations are defined in the Methods section. Source data are provided as a Source data file.

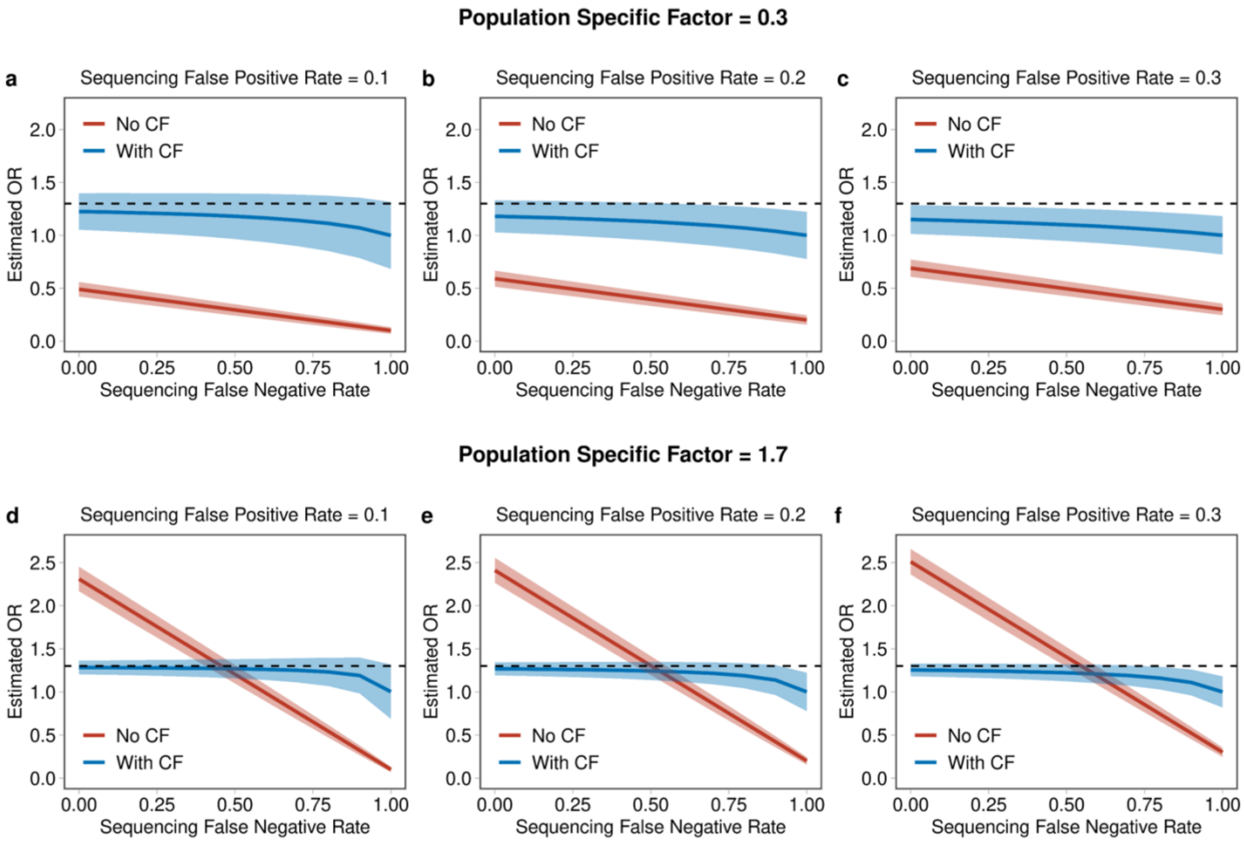

Supplementary Figure 2: Simulated effects of combined SFN rate, SFP rate, and high PSF on estimated OR. Probability of mutation (*P*) was calculated as a function of the cumulative minor allele frequency for a single gene ($CMAF_{Gene}$: set at 0.05), the true effect size of association (OR), and the 3 major association biases (SFN, SFP, and PSF) according to equation 2. The simulations kept the true OR fixed at 1.3 (black dashed lines) while varying SFN rate (0 to 1), SFP rate (0.1, 0.2, and 0.3), and high PSF (0.3 and 1.7). A mean estimated OR was calculated for each *P* (corresponding to a unique combination of the 3 association biases) and plotted as a function of the combined biases (**a-f; red lines**) across 100,00 simulations. CF values were calculated as the ratio of the *P* for each estimated OR and the original $CMAF_{Gene}$ according to equation 3. Each CF was then used to adjust the $CMAF_{Gene}$ to calculate an adjusted *P* (P^*) according to equation 4. Using P^*, a CF-adjusted mean estimated OR was plotted as a function of the combined biases (**a-f; blue lines**) across 100,000 simulations. All shaded regions correspond to the 95% confidence interval for the mean estimated OR. OR indicates odds ratio, CF indicates correction factor, SFN indicates sequencing false negatives, SFP indicates sequencing false positives, PSF indicates population-specific factor. Equations are defined in the Methods section. Source data are provided as a Source data file.

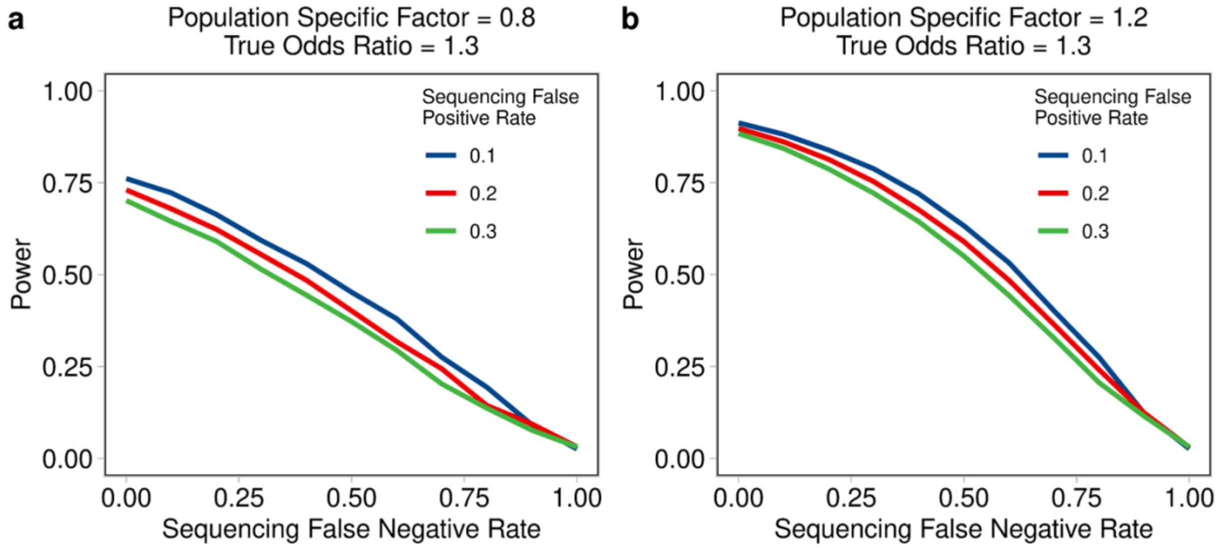

Supplementary Figure 3: Power to detect gene-based association signals in the presence of SFN rate, SFP rate, and PSF. CF-adjusted power curves were generated under the simultaneous effect of SFN rate (0 to 1), SFP rate (0.1(**blue line**), 0.2 (**red line**), 0.3 (**green line**)), and PSF (0.8 for **a** and 1.2 for **b**) at a fixed true odds ratio value of 1.3. Power for every value of SFN, SFP, and PSF was calculated as the proportion of all simulations with a *P*-value < 0.05, which was calculated as described in the Methods section. Source data are provided as a Source data file.

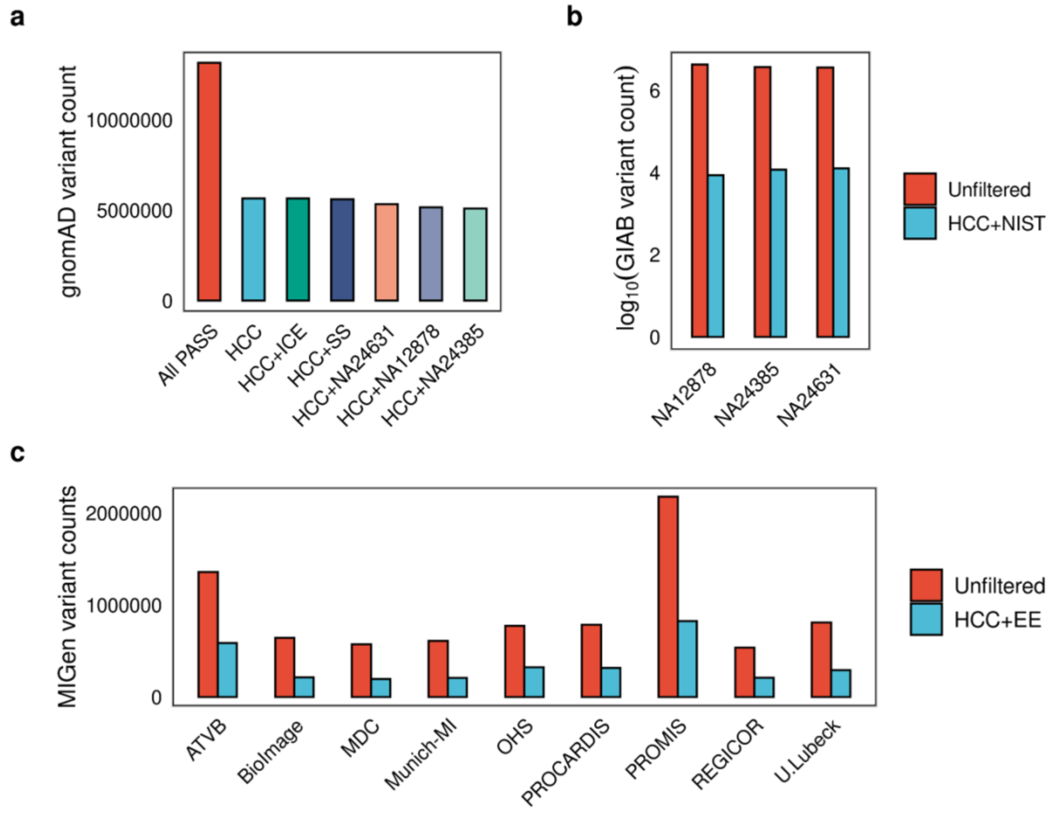

Supplementary Figure 4: Number of variant sites remaining in (a) gnomAD, (b) GIAB, and (c) MIGen after intersection with different region intervals. For (**a**), HCC+ICE: intersection sites between high-coverage coding sites (defined in the Methods section) and the ICE-capture exome enrichment kit; HCC+SS: intersection between HCC sites and SureSelect (SS) Human All Exon v.2 Kit; HCC+NA24631: intersection between HCC and NIST high-confidence sites for GIAB sample NA24631; HCC+NA12878: intersection between HCC and NIST high-confidence sites for GIAB sample NA12878; HCC+NA24385: intersection between HCC and NIST high-confidence sites for GIAB sample NA24631. For (**b**), HCC+NIST: intersection between HCC sites and each respective NIST high-confidence interval site for each GIAB sample. For (**c**), HCC+EE: intersection between HCC sites and intervals from the exome enrichment (EE) kit used for a given MIGen cohort. HCC indicates high coverage coding sites, SS indicates SureSelect, NIST indicates National Institute of Standards and Technology, MIGen indicates Myocardial Infarction Genetics, gnomAD indicates genome Aggregation Database, and GIAB indicates Genome In A Bottle. Source data are provided as a Source data file.

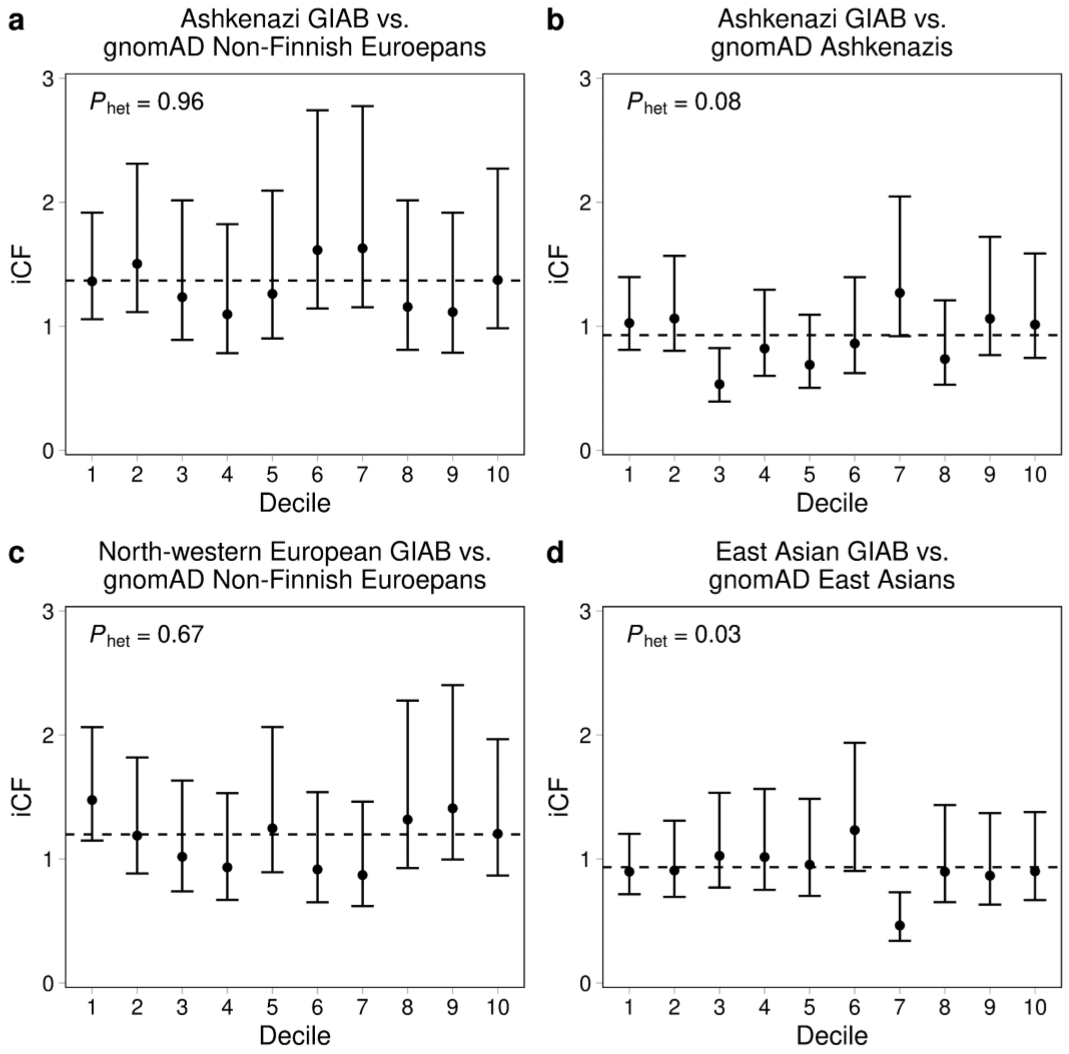

Supplementary Figure 5: GIAB iCF values for gene groups stratified by decile of gene constraint score. Ethnic comparisons include the Ashkenazi GIAB sample (NA24385) with gnomAD non-Finnish Europeans and gnomAD Ashkenazis (**a** and **b**), the North-western European GIAB sample (NA12878) with gnomAD Non-Finnish Europeans (**c**), and the East Asian GIAB sample (NA24631) with gnomAD East Asians (**d**). All genes were grouped by decile according to their missense constraint metric (Lek *et al.*, 2016[16]) across 13,470 genes for **(a)**, 13,463 genes for **(b)**, 13,841 genes for **(c)**, and 14,128 genes for **(d)**. Points represent the iCF values that were calculated all genes in a given decile as ratio of the sum of per-individual observed allele counts (OAC) to the sum of per-individual expected allele counts in gnomAD (EAC) according to equation 5. Error bars depict 95% confidence intervals of the iCF and dashed lines represent the iCF across all deciles (i.e. exome-wide iCF). *P*-values for heterogeneity (P_{het}) across deciles were calculated with a fixed effect meta-analysis as described in the Methods section. Any $P_{het} < 0.0125$ (0.05/4) was considered significant after multiple hypothesis testing. iCF indicates individual correction factor, GIAB indicates Genome In A Bottle, and gnomAD indicates genome aggregation database. Equations are defined in the Methods section. Source data are provided as a Source data file.

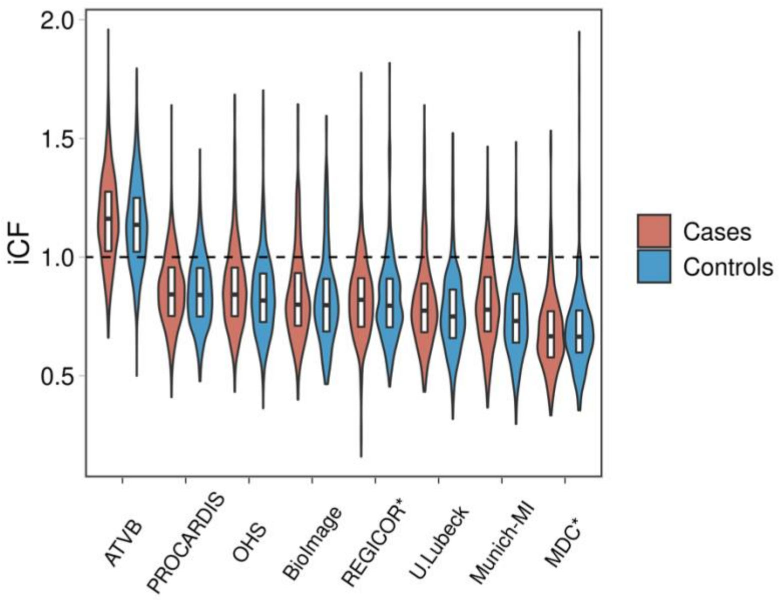

Supplementary Figure 6: iCF values for cases and controls across all discovery MIGen cohorts. Distribution of iCF values are shown for case (**red**) and control (**blue**) participants across 8 MIGen cohorts. iCF values were calculated as the exome-wide ratio of the sum of per-individual observed allele counts (OAC) to the sum of per-individual expected allele counts in gnomAD (EAC) according to equation 5. Violins demonstrate the spread of iCF values. The horizontal line in each boxplot indicate the median iCF value while the top and bottom lines represent the 75th and 25th percentiles of the iCF distribution, respectively. Length of boxplot represents the inter-quartile range of iCF values. Cohorts marked with an asterisk were found to have a significantly different (P<0.05) iCF distribution between cases and controls after adjusting for sex and the first 20 principal components of ancestry (age was not available as a phenotype in MIGen). The dashed line represents an equal total allele count between MIGen cases or controls versus gnomAD non-Finnish Europeans. iCF indicates individual correction factor. Equations are defined in the Methods section. Source data are provided as a Source data file.

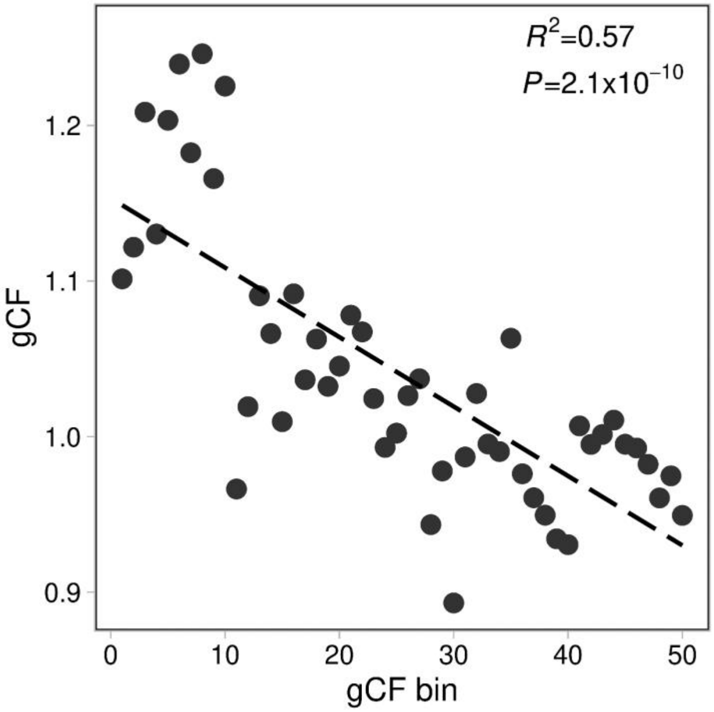

Supplementary Figure 7: gCF values across 50 gene bins derived from 3,352 healthy MIGen controls. gCF values were computed as the ratio of the sum of the OAC to the sum of iCF-adjusted EAC across 1) all individuals and 2) all genes that were organized into one of 50 gene bins according to equation 7. Gene bins were ascertained according to quintile of iCF-adjusted EAC and decile of *P*-value obtained from a rare variant association test (using burden of rare pathogenic alleles) conducted in the ranking cohort, consisting of the remaining 2,730 healthy control participants in MIGen as "cases" and gnomAD non-Finnish Europeans as controls. R^2 and *P*-value was evaluated through a linear regression model that evaluated the variance explained in gCF by the gCF bin number. gCF indicates gene correction factor. Equations are defined in the Methods section. Source data are provided as a Source data file.

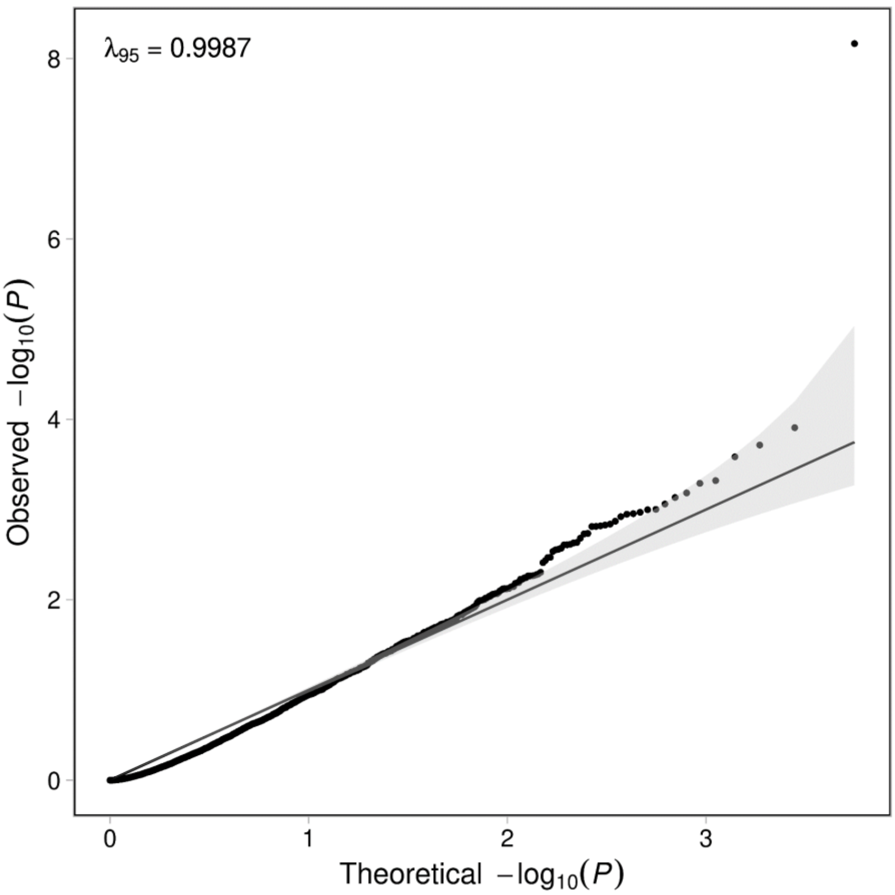

Supplementary Figure 8: Calibration of gene-based test statistics using quality-by-depth (QD) scores for rare synonymous SNVs according to the TRAPD algorithm for 3,352 healthy control participants in MIGen. A quantile-quantile plot is shown that demonstrates the distribution of gene-based test statistics generated from rare synonymous SNVs using the TRAPD method. Genomic inflation factors at the 95th percentile of gene-based test statistics (λ_{95}) were calculated based on varying percentiles of QD scores for rare synonymous SNVs in 3,352 healthy controls from MIGen, which were used as “cases” and non-Finnish Europeans in gnomAD, which were used as controls. The distribution gene-based test statistics that are shown correspond to rare synonymous SNVs then fell into the 94th percentile of QD scores in MIGen healthy controls and 90th percentile of QD scores in gnomAD, which collectively achieved a λ_{95} closest to 1. The solid black line indicates an expected uniform distribution of *P*-values under the null and the shaded region represents the 95% confidence interval of the expected uniform distribution of *P*-values. $\log_{10}(P)$ refers to the log base 10 of a gene-based *P*-value. Source data are provided as a Source data file.

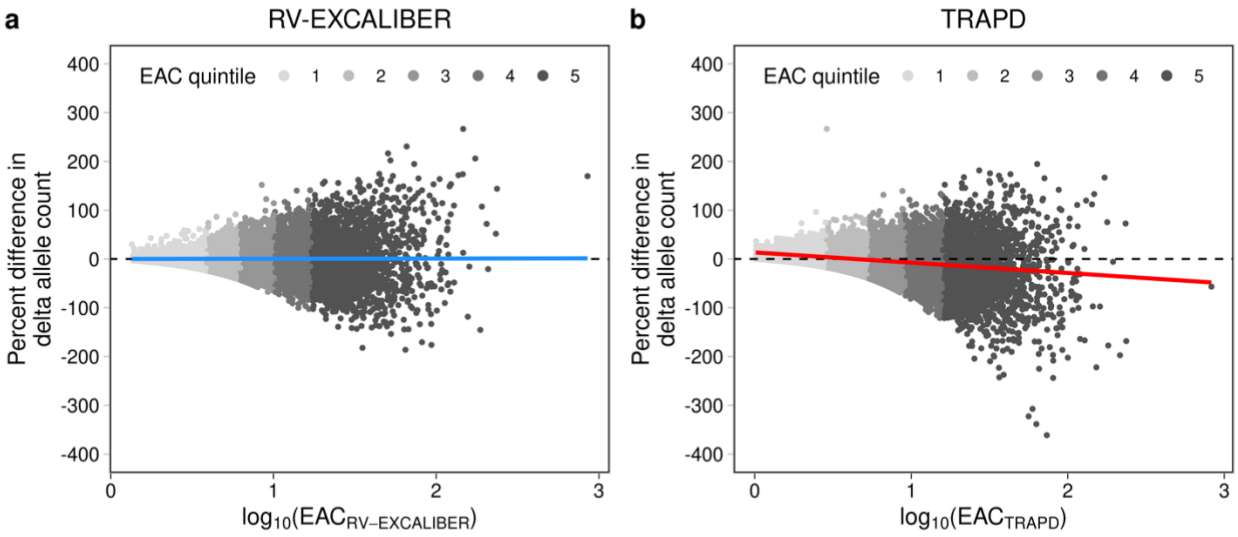

Supplementary Figure 9: Dispersion of delta counts generated using RV-EXCALIBER or TRAPD across the spectrum of EAC for 3,352 healthy control participants in MIGen. Delta allele counts (*D*) were calculated according to equations 8 and 9 as the per-gene difference between the observed allele count (OAC) and the expected allele count in gnomAD (EAC) across 3,352 healthy control participants from MIGen and gnomAD, respectively. A total of 10,788 genes which had OAC and EAC ≥1 in both methods were included and were ascertained for rare pathogenic alleles. EAC from RV-EXCALIBER **(a)** are adjusted by the both the iCF and gCF according to equation 8, while the OAC and EAC for TRAPD **(b)** were obtained from percentile cut-offs of variant-level QD scores for the same 3,352 healthy control participants from MIGen and gnomAD (see main text results). The EAC for either method was $\log_{10}$ transformed and assigned to quintiles. A regression line representing the mean percent difference was then plotted for both RV-EXCALIBER (**blue line**) and TRAPD (**red line**). Horizontal dashed lines indicate zero percent difference between OAC and EAC. Equations are defined in the Methods section. Source data are provided as a Source data file.

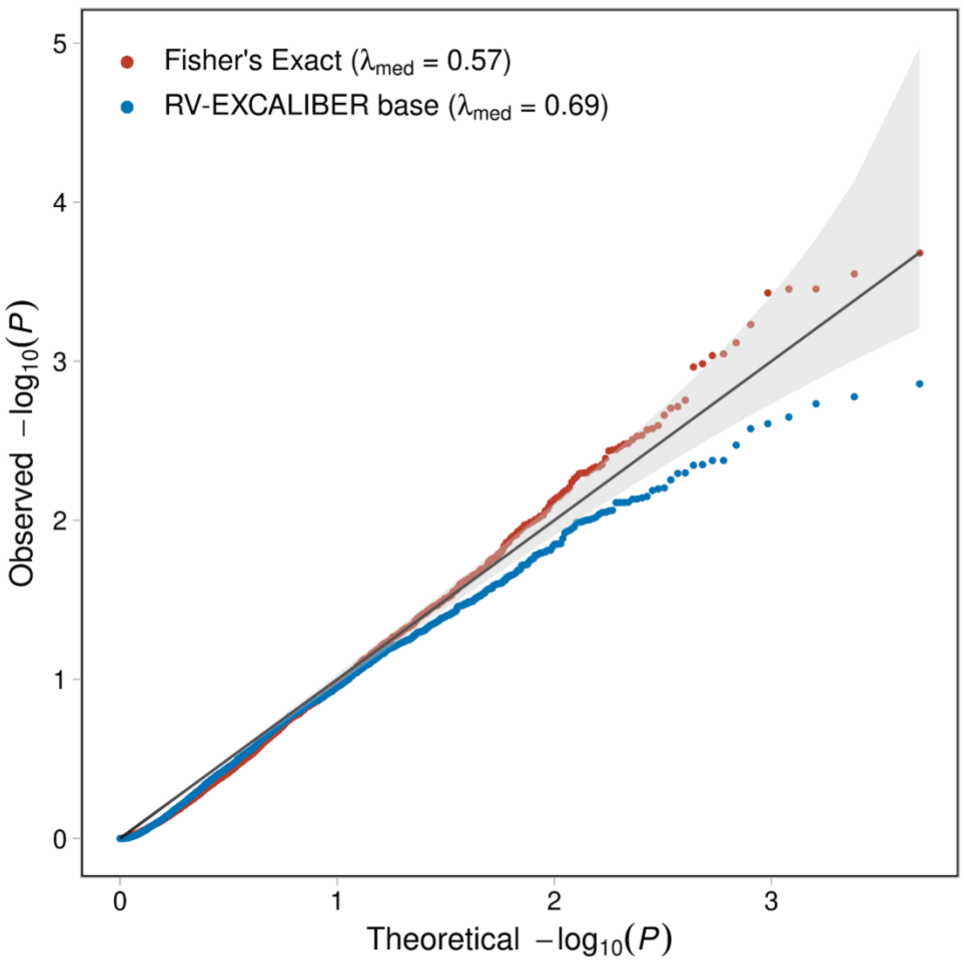

Supplementary Figure 10: Quantile-quantile plots for gene-based test statistics generated using RV-EXCALIBER base and a Fisher's Exact test for 3,352 healthy control participants in MIGen. RV-EXCALIBER base only accounts for linkage disequilibrium between rare variants and does not incorporate iCF or iCF and gCF-adjustments. A total of 4,815 protein-coding genes with OAC ≥1 in MIGen and EAC ≥10 in gnomAD non-Finnish Europeans were used to conduct a gene burden test using rare pathogenic alleles according to both RV-EXCALIBER base (**blue points**) and a Fisher's Exact (**red points**) tests. Since RV-EXCALIBER provides 1-sided *P*-values, a 1-sided Fisher's Exact test was also used to evaluate enrichment of OAC for a given gene. A total of 3,352 healthy control participants were used as "cases" in MIGen and gnomAD non-Finnish Europeans were used as controls. Genomic inflation estimates were evaluated at the median (λ_{med}). The solid black line indicates an expected uniform distribution of *P*-values under the null and the shaded region represents the 95% confidence interval of the expected uniform distribution of *P*-values. $\log_{10}(P)$ refers to the log base 10 of a gene-based *P*-value. Source data are provided as a Source data file.

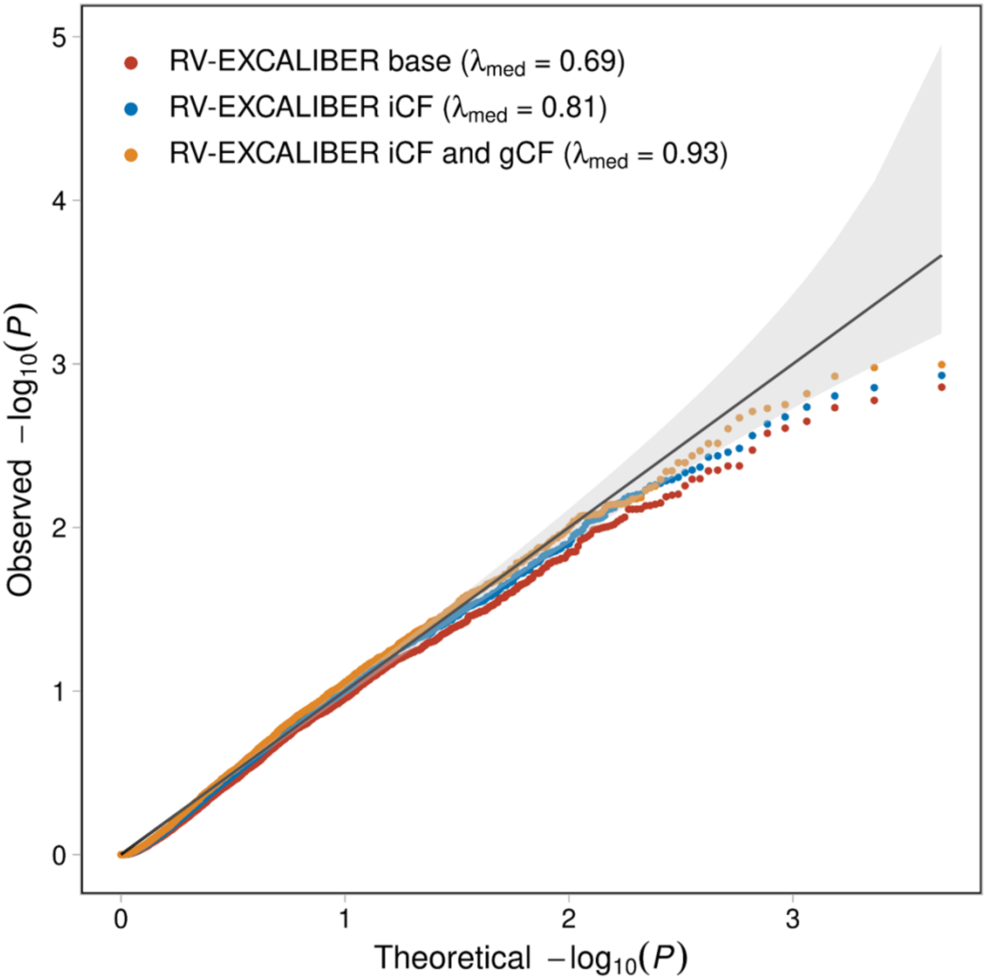

Supplementary Figure 11: Quantile-quantile plots for gene-based test statistics generated using RV-EXCALIBER base, RV-EXCALIBER iCF and RV-EXCALIBER iCF and gCF (i.e. fully adjusted model) for 3,352 healthy control participants in MIGen. RV-EXCALIBER base (**red points**) only accounts for linkage disequilibrium between rare variants does not incorporate iCF or iCF and gCF-adjusted EAC, which are used in RV-EXCALIBER iCF (**blue points**) and RV-EXCALIBER iCF and gCF (**gold points**), respectively. A total of 4,815 protein-coding genes with OAC ≥1 in MIGen and EAC ≥10 in gnomAD non-Finnish Europeans were used to conduct a rare variant association test. A gene burden test using rare pathogenic alleles was implemented using 3,352 healthy control participants as "cases" in MIGen and gnomAD non-Finnish Europeans as controls. The remaining 2,730 healthy control participants were used as the ranking cohort to generate gCF values. Genomic inflation estimates were evaluated at the median (λ_{med}). The solid black line indicates an expected uniform distribution of *P*-values under the null and the shaded region represents the 95% confidence interval of the expected uniform distribution of *P*-values. $\log_{10}(P)$ refers to the log base 10 of a gene-based *P*-value. Source data are provided as a Source data file.

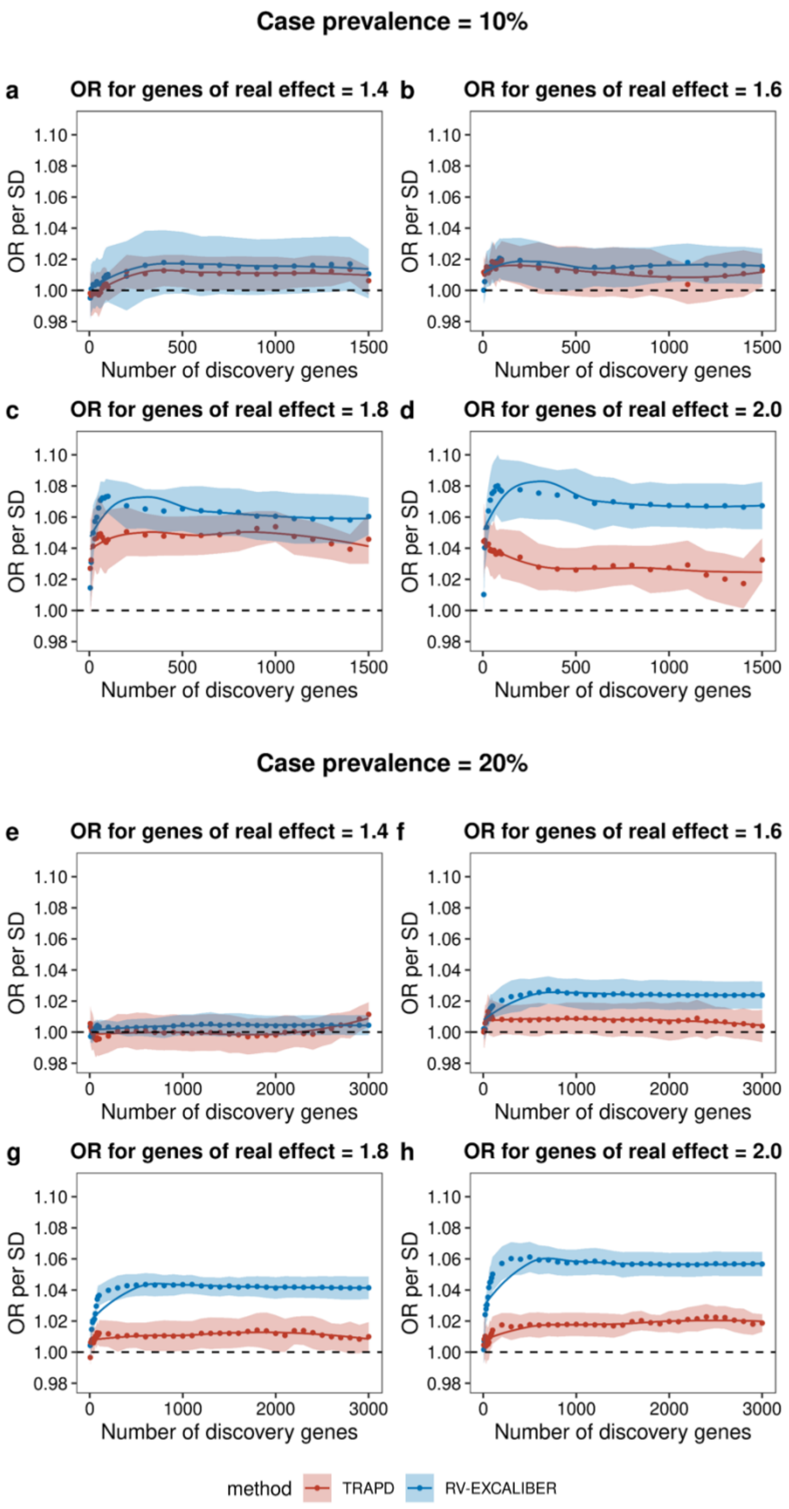

Supplementary Figure 12: Predictive effect of TRAPD and RV-EXCALIBER-derived RVGRS on simulated case status in the UK Biobank. Benchmark simulations were performed

by conducting discovery gene-based rare variant association analysis using the TRAPD (**red points**) and RV-EXCALIBER (**blue points**) methods on cases that were ascertained according to a probability-based sampling approach, where probability of being a case was ascertained according to a pre-assigned regression coefficient for 100 "genes of real effect" (gre) and the delta allele count for the gre for a given individual according to equation 14. A total of 10 case sampling simulations were performed across 2 disease prevalence parameters: 10% (**a-d**); 20% (**e-h**) and across 4 fixed effect sizes for the gre, corresponding to odds ratios of 1.4 (**a** and **e**), 1.6 (**b** and **f**) 1.8 (**c** and **g**), and 2.0 (**d** and **h**). Each point indicates the mean odds ratio per 1 SD change in RVGRS on simulated case status, where the mean odds ratio was ascertained for a given number of discovery genes across 10 case simulations for each case prevalence and gre effect size parameter. Solid lines represent locally weighted smoothing. Shaded regions correspond to bootstrapped 95% confidence intervals of the mean odds ratio. Dashed line represents a line of no effect. OR indicates odds ratio, SD indicates standard deviation. Equations are defined in the Methods section. Source data are provided as a Source data file.

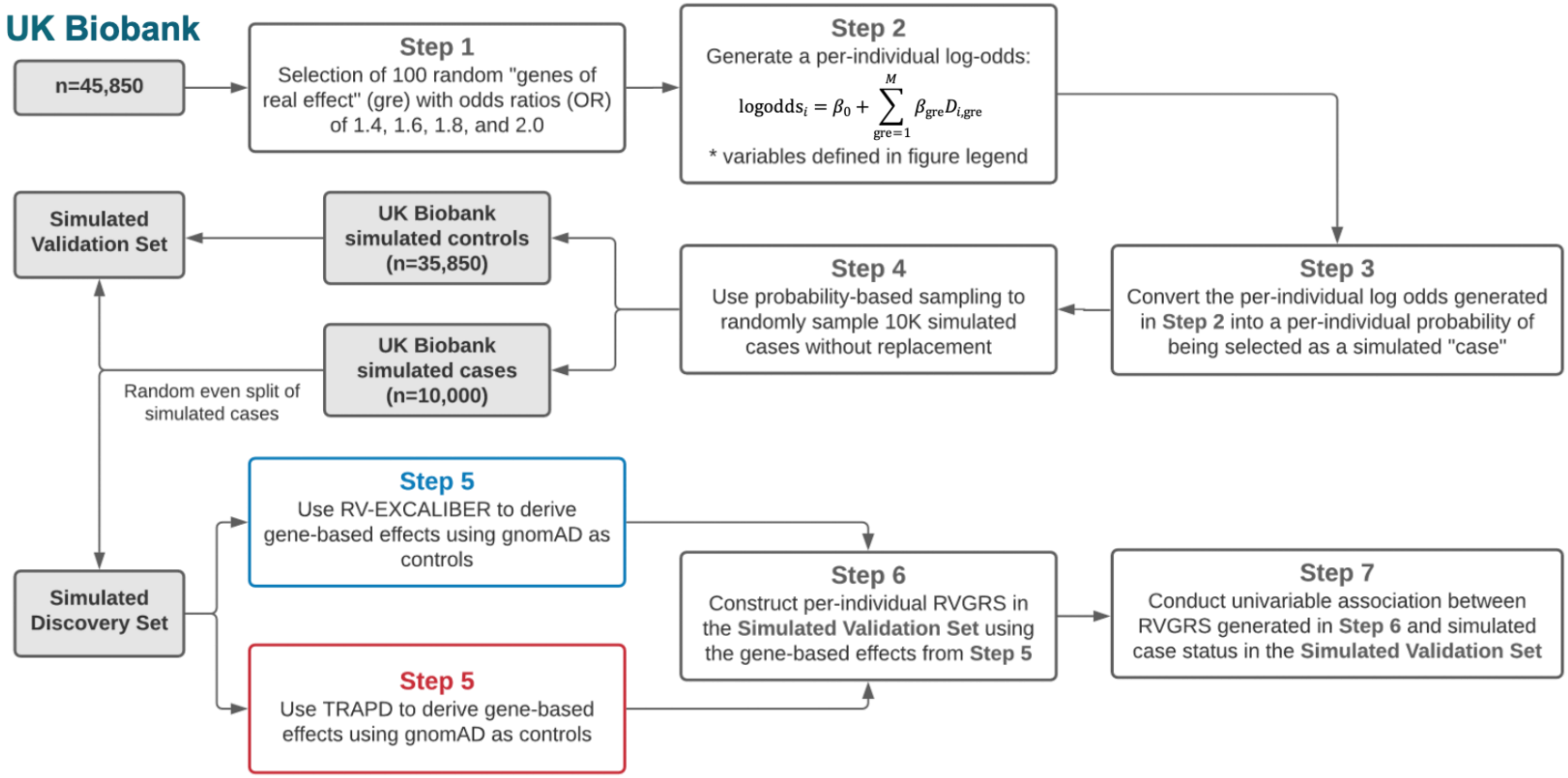

Supplementary Figure 13: Workflow for benchmark simulations to assess the predictive power of the RV-EXCALIBER and TRAPD frameworks. For Step 2, $\beta_0$refers to the prevalence of cases in a specific simulation, β_{gre} refers to the $\log(\text{OR}_{\text{gre}})$ outlined in Step 1, and $D_{i,\text{gre}}$ refers to the per-individual delta allele count for the gre. A total of 10 simulations were performed per case prevalence parameter (10% and 20%) per effect size for the gre (odds ratio = 1.4, 1.6, 1.8, 2.0). In this flow chart, a simulated case prevalence of ~20% (i.e. 10,000/45,850) is shown for illustration. OR indicates odds ratio, gre indicates “genes of real effect”, gnomAD indicates genome Aggregation Database, and RVGRS indicates rare variant genetic risk score.

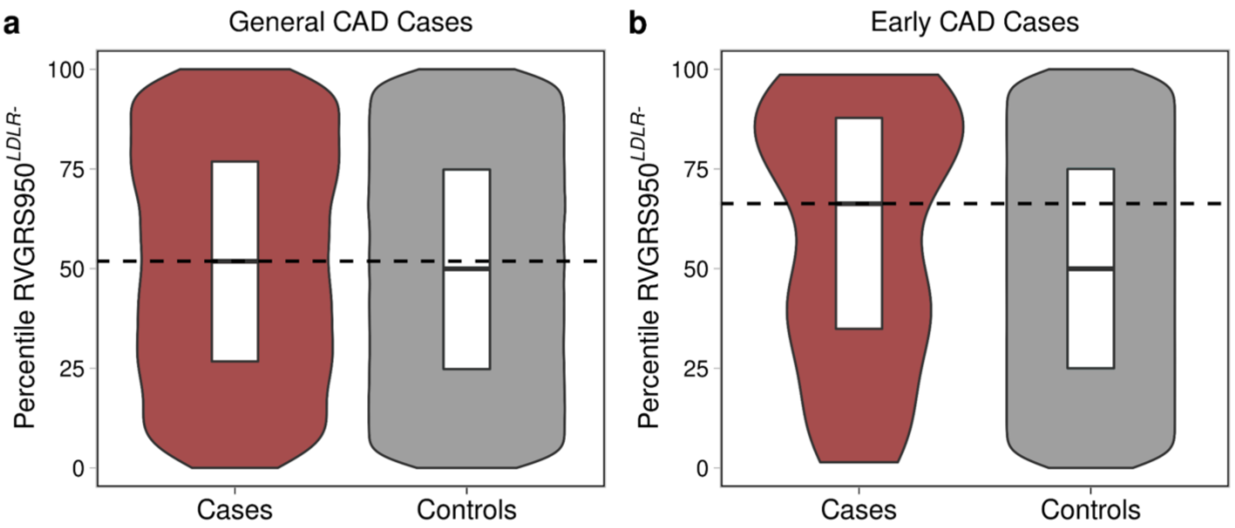

Supplementary Figure 14: Distribution of RVGRS950*LDLR-* among CAD-free controls, general CAD cases, and early CAD cases in the UK Biobank. Distribution of RVGRS950*LDLR-* was evaluated for UK Biobank control participants and UK Biobank case participants with general CAD (i.e. CAD irrespective of age of onset) **(a)**, as well as UK Biobank control participants and UK Biobank participants with early CAD (i.e. males ≤ 40 and females ≤ 45) **(b)**. The violins demonstrate the spread of RVGRS950*LDLR-* distribution for CAD cases (**red**) and CAD-free controls (**grey**). The horizontal line in each boxplot indicate the median RVGRS950*LDLR-* value while the top and bottom lines represent the 75th and 25th percentiles of the RVGRS950 distribution, respectively. Length of boxplot represents the inter-quartile range of RVGRS950*LDLR-* values. The dashed lines represent where the median of each respective case group falls in the control distribution. RVGRS950*LDLR-* indicates rare variant genetic risk score from the top 950 discovery genes (excluding *LDLR*), and CAD indicates coronary artery disease. Source data are provided as a Source data file.

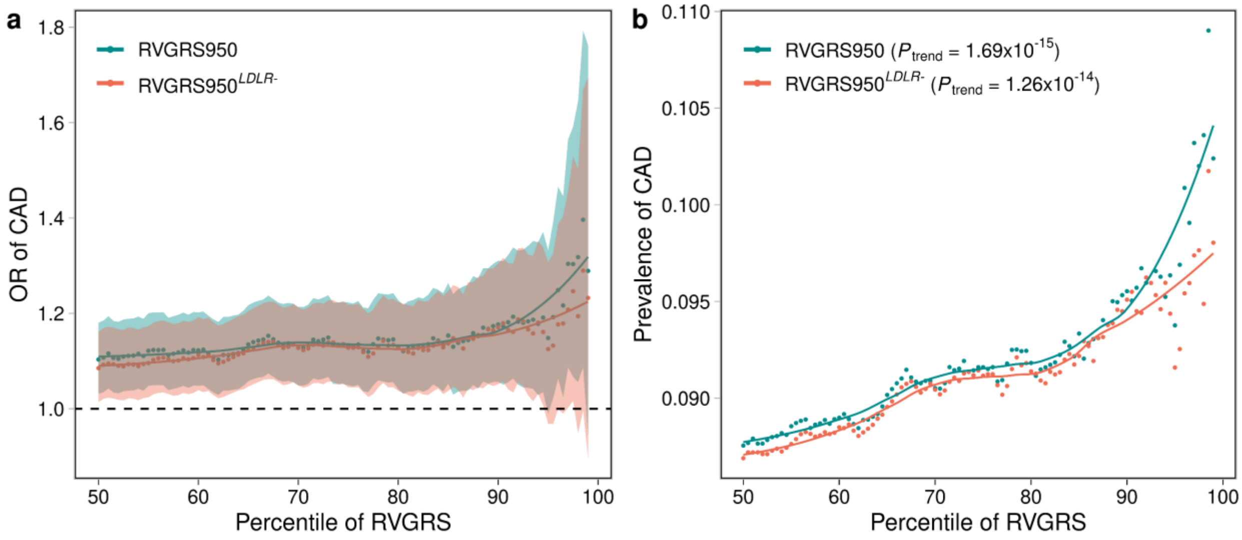

Supplementary Figure 15: Predictive effect and prevalence of CAD across increasing RVGRS950 and RVGRS950*$^{LDLR-}$* percentile groupings in the UK Biobank. (a) Each point represents the odds ratios of CAD that were calculated across the top 100 percentile groupings (50th to 99.9th in intervals of 0.05%) and are in reference to the remaining distribution of RVGRS950 (**green points**) or RVGRS950^{LDLR-} (**orange points**) (e.g. the 90th percentile refers to odds of CAD for individuals in the top 10% of RVGRS950 or RVGRS950^{LDLR-} relative to the remaining 90% of the population). Shaded regions correspond to the 95% confidence interval of this odds ratio. Odds ratios were adjusted for age, age^2, sex, and the first 20 principal components of ancestry. **(b)** Each point represents the CAD prevalence that was calculated as the proportion of individuals with CAD across each percentile threshold of RVGRS950 (**green points**) or RVGRS950^{LDLR-} (**orange points**). Significance for change in CAD prevalence across increasing percentile of RVGRS was evaluated using the Cochrane-Armitage test for trend. Solid lines for **(a)** and **(b)** represent locally weighted smoothing. OR indicates odds ratio, RVGRS950 refers to rare variant genetic risk score from the top 950 discovery genes, RVGRS950^{LDLR-} indicates rare variant genetic risk score from the top 950 discovery genes (excluding *LDLR*), and CAD indicates coronary artery disease. Source data are provided as a Source data file.

Supplementary Tables

Supplementary Table 1: Baseline and sequencing characteristics for individual cohorts used from the MIGen consortium.

MIGen Cohort	dbGaP study accession (link)	Ethnicity	% Reported Male	Sequencing platform	Exome enrichment kit	Cases / Controls retained after QC (N)	Cases / Controls retained after QC (%)
Italian Atherosclerosis Thrombosis and Vascular Biology (ATVB)	phs000814.v1.p1 [https://www.ncbi.nlm.nih.gov/projects/gap/cgi-bin/study.cgi?study_id=phs000814.v1.p1]	European	89	HiSeq 2000 and 2500	SureSelect Human All Exon v.2 Kit	1784 / 1719	98 / 97
Malmö Diet and Cancer Study (MDC)	phs001101.v1.p1 [https://www.ncbi.nlm.nih.gov/projects/gap/cgi-bin/study.cgi?study_id=phs001101.v1.p1]	European	55	HiSeq 2000 and 2500	ICE Capture Reagent	518 / 522	96 / 97
BioImage Study (BioImage)	phs001058.v1.p1 [https://www.ncbi.nlm.nih.gov/projects/gap/cgi-bin/study.cgi?study_id=phs001058.v1.p1]	European	61	HiSeq 2000 and 2500	ICE Capture Reagent	128 / 269	84 / 77
University of Lubeck (U. Lubeck)	phs000990.v1.p1 [https://www.ncbi.nlm.nih.gov/projects/gap/cgi-bin/study.cgi?study_id=phs000990.v1.p1]	European	61	HiSeq 2000 and 2500	ICE Capture Reagent	799 / 859	92 / 96
German Heart Centre in Munich (Munich-MI)	phs000916.v1.p1 [https://www.ncbi.nlm.nih.gov/projects/gap/cgi-bin/study.cgi?study_id=phs000916.v1.p1]	European	65	HiSeq 2000 and 2500	ICE Capture Reagent	386 / 394	97 / 99
Registre Gironi del Cor (REGICOR)	phs000902.v1.p1 [https://www.ncbi.nlm.nih.gov/projects/gap/cgi-bin/study.cgi?study_id=phs000902.v1.p1]	European	77	HiSeq 2000 and 2500	SureSelect Human All Exon v.2 Kit	357 / 390	93 / 97
Precocious Coronary Artery Disease Study (PROCARDIS)	phs000883.v1.p1 [https://www.ncbi.nlm.nih.gov/projects/gap/cgi-bin/study.cgi?study_id=phs000883.v1.p1]	European	65	HiSeq 2000 and 2500	SureSelect Human All Exon v.2 Kit	983 / 959	95 / 94
Ottawa Heart Study (OHS)	phs000806.v1.p1 [https://www.ncbi.nlm.nih.gov/projects/gap/cgi-bin/study.cgi?study_id=phs000806.v1.p1]	European	67	HiSeq 2000 and 2500	SureSelect Human All Exon v.2 Kit	955 / 970	97 / 98
Pakistan Risk of Myocardial Infarction Study (PROMIS)	phs000917.v1.p1 [https://www.ncbi.nlm.nih.gov/projects/gap/cgi-bin/study.cgi?study_id=phs000917.v1.p1]	South Asian	83	HiSeq 2000 and 2500	ICE Capture Reagent and SureSelect Human All Exon v.2 Kit	2946 / 3708	97 / 92

Supplementary Table 2: Ethnicities in gnomAD used to generate the EAC for a given GIAB sample. GIAB indicates Genome In a Bottle, gnomAD indicates genome Aggregation Database, EAC indicates expected allele count.

ID of GIAB sample	Ethnicity of GIAB sample	Ethnicity in gnomAD used to determine the EAC
NA12878	North-western European	Non-Finnish European
NA24631	Ease Asian	East Asian
NA24385	Ashkenazi Jewish	Non-Finnish European
		Ashkenazi Jewish

Supplementary Table 3: iCF values for every GIAB vs. gnomAD ethnicity across 4 allele frequency bins. AF indicates allele frequency, GIAB indicates Genome In A Bottle, gnomAD indicates genome Aggregation Database, iCF indicates individual correction factor, CI indicates confidence interval.

AF bin	GIAB ethnicity	gnomAD population	iCF	95% CI
**0-0.01**	North-western European	Non-Finnish European	1.20	1.07-1.36
	East Asian	East Asian	0.93	0.84-1.04
	Ashkenazi	Ashkenazi	0.93	0.84-1.04
	Ashkenazi	Non-Finnish European	1.37	1.22-1.55
**0.01-0.05**	North-western European	Non-Finnish European	0.93	0.86-1.01
	East Asian	East Asian	1.00	0.92-1.11
	Ashkenazi	Ashkenazi	0.93	0.86-1.01
	Ashkenazi	Non-Finnish European	0.98	0.91-1.08
**0.05-0.25**	North-western European	Non-Finnish European	1.03	0.99-1.06
	East Asian	East Asian	0.98	0.95-1.01
	Ashkenazi	Ashkenazi	0.99	0.96-1.02
	Ashkenazi	Non-Finnish European	0.98	0.95-1.01
**0.25-0.50**	North-western European	Non-Finnish European	1.00	0.99-1.02
	East Asian	East Asian	0.97	0.96-0.99
	Ashkenazi	Ashkenazi	1.00	0.99-1.02
	Ashkenazi	Non-Finnish European	1.02	1.01-1.04

Supplementary Table 4: Median iCF values for healthy control participants across 8 MIGen cohorts. iCF indicates individual correction factor and IQR indicates inter-quartile range.

MIGen cohort	Median iCF (IQR)
ATVB	1.135 (0.909-1.363)
PROCARDIS	0.841 (0.636-1.045)
OHS	0.817 (0.613-1.022)
BioImage	0.797 (0.576-1.019)
U. Lubeck	0.795 (0.591-1.000)
Munich-MI	0.750 (0.545-0.954)
REGICOR	0.731 (0.525-0.936)
MDC	0.664 (0.487-0.841)

Supplementary Table 5: Predictive effect of iCF distribution in MIGen control versus case participants. All odds ratios and *P*-values were calculated using a multivariable logistic regression model that was adjusted for sex and the first 20 principal components of ancestry. OR indicates odds ratio and CI indicates confidence interval.

**MIGen cohort**	**OR (95% CI)***	***P*-value**
ATVB	0.83 (0.56-1.25)	0.381
PROCARDIS	0.75 (0.40-1.40)	0.370
OHS	0.73 (0.37-1.44)	0.364
BioImage	0.86 (0.17-4.49)	0.857
U. Lubeck	0.96 (0.48-1.92)	0.911
Munich-MI	0.39 (0.14-1.10)	0.0745
REGICOR‡	0.33 (0.12-0.92)	0.0352
MDC‡	2.44 (1.01-5.96)	0.0492

*OR is expressed as controls relative to cases
‡ Nominal significance (P<0.05)

Supplementary Table 6: Gene-set enrichment for nominally associated (P<0.05) discovery genes from RV-EXCALIBER. Fold enrichment was calculated according to the ratio of the proportion of target genes (i.e. discovery genes with *P*<0.05) to the proportion of all genes (i.e. all discovery genes) in a given GO biological process. Enrichment *P*-values were calculated from a minimum hypergeometric test statistic. GO indicates gene ontology.

GO term	Description	Fold enrichment	*P*-value
GO:0009605	response to external stimulus	1.79	$8.46x10^{-6}$
GO:0042221	response to chemical	1.48	$5.02x10^{-5}$
GO:0050896	response to stimulus	1.31	$6.99 x10^{-5}$
GO:0010033	response to organic substance	1.52	$1.13x10^{-4}$
GO:0045630	positive regulation of T-helper 2 cell differentiation	20.47	$1.16 x10^{-4}$
GO:0045628	regulation of T-helper 2 cell differentiation	20.47	$1.16 x10^{-4}$
GO:0060059	embryonic retina morphogenesis in camera-type eye	20.47	$1.16 x10^{-4}$
GO:1903409	reactive oxygen species biosynthetic process	6.82	$1.47 x10^{-4}$
GO:0006950	response to stress	1.4	$4.74 x10^{-4}$
GO:1901700	response to oxygen-containing compound	1.58	$5.39 x10^{-4}$
GO:0007616	long-term memory	6.4	$7.56 x10^{-4}$
GO:0009894	regulation of catabolic process	1.75	$9.26 x10^{-4}$
GO:0006809	nitric oxide biosynthetic process	8.19	$9.30 x10^{-4}$
GO:0042592	homeostatic process	1.52	$9.47 x10^{-4}$

Supplementary Table 7: Predicitve effect of RVGRS950 and RVGRS950*LDLR-* on CAD across tertiles of FRS in the UK Biobank. All odds ratios and *P*-values were calculated using a multivariable logistic regression model that was adjusted for age, age^2, sex, and the first 20 principal components of ancestry. FRS indicates Framingham risk score, OR indicates odds ratio, SD indicates standard deviation, and CI indicates confidence interval.

FRS tertile	OR per SD (95% CI)	*P*-value	$P_{interaction}$
RVGRS950			
1	1.16 (1.06-1.26)	$7.3x10^{-4}$	0.018
2	1.10 (1.04-1.17)	$1.6x10^{-3}$	
3	1.04 (0.99-1.09)	0.13	
RVGRS950*LDLR-*			
1	1.15 (1.05-1.25)	$2.0x10^{-3}$	0.024
2	1.09 (1.03-1.16)	$4.0x10^{-3}$	
3	1.03 (0.98-1.08)	0.22	

Supplementary Table 8: Predictive effect of CVGRS on tertiles on CAD across tertile of FRS in the UK Biobank. All odds ratios and *P*-values were calculated using a multivariable logistic regression model that was adjusted for age, age^2, sex, and the first 20 principal components of ancestry. FRS indicates Framingham risk score, OR indicates odds ratio, SD indicates standard deviation, and CI indicates confidence interval.

FRS tertile	OR per SD (95% CI)	*P*-value	$P_{interaction}$
1	1.36 (1.24-1.49)	$2.1x10^{-11}$	0.74
2	1.26 (1.19-1.34)	$3.2x10^{-14}$	
3	1.30 (1.25-1.37)	$< 2.2x10^{-16}$	

Supplementary Table 9: Net-reclassification improvement index when predicting CAD status after incorporating RVGRS950 and RVGRS950^{LDLR-} to risk models. Reference and test models were evaluated using a multivariable logistic regression model that was adjusted for age, age^2, sex, and the first 20 principal components of ancestry. NRI index and P-value were ascertained based on the proportion of CAD cases and CAD-free controls that were correctly re-classified using the test models (relative to the reference models). FRS indicates Framingham risk score, CVGRS indicates common variant genetic risk score, RVGRS indicates rare variant genetic risk score, NRI indicates net-reclassification improvement.

Reference model	Test model	NRI index[a]	*P*-value for NRI index
RVGRS950			
FRS	FRS + RVGRS	0.0548	1.1×10^{-3}
CVGRS	CVGRS + RVGRS	0.0541	1.3×10^{-3}
FRS + CVGRS	FRS + CVGRS + RVGRS	0.0558	9.2×10^{-4}
RVGRS950^{LDLR-}			
FRS	FRS + RVGRS	0.0435	9.7×10^{-3}
CVGRS	CVGRS + RVGRS	0.436	9.6×10^{-3}
FRS + CVGRS	FRS + CVGRS + RVGRS	0.0451	7.4×10^{-3}

[a] NRI index is comparing the test model to the reference model

Supplementary references

1. Karczewski, K. J. *et al.* Variation across 141,456 human exomes and genomes reveals the spectrum of loss-of-function intolerance across human protein-coding genes. *bioRxiv* 531210 (2019) doi:10.1101/531210.
2. Zook, J. M. *et al.* Integrating human sequence data sets provides a resource of benchmark SNP and indel genotype calls. *Nat. Biotechnol.* **32**, 246–251 (2014).
3. Zook, J. M. *et al.* An open resource for accurately benchmarking small variant and reference calls. *Nat. Biotechnol.* **37**, 561–566 (2019).
4. NCBI. Decrypting and Extracting Data. https://www.ncbi.nlm.nih.gov/books/NBK63512/.
5. Tennessen, J. a *et al.* Evolution and functional impact of rare coding variation from deep sequencing of human exomes. *Science* **337**, 64–9 (2012).
6. Heart, N. Loss-of-Function Mutations in APOC3, Triglycerides, and Coronary Disease. *N. Engl. J. Med.* 1–10 (2014) doi:10.1056/NEJMoa1307095.
7. Do, R. *et al.* Exome sequencing identifies rare LDLR and APOA5 alleles conferring risk for myocardial infarction. *Nature* **518**, 102–6 (2015).
8. Garrison, E. P. vcflib: A C++ library for parsing and manipulating VCF files. https://github.com/vcflib/vcflib#vcflib.
9. Li, Heng; Handsaker , B; Danecek, Petr; McCarthy , S; Marshall, J. BCFtools. https://github.com/samtools/bcftools.
10. Chang, C. C. *et al.* Second-generation PLINK: rising to the challenge of larger and richer datasets. *Gigascience* **4**, 7 (2015).
11. Yang, J., Lee, S. H., Goddard, M. E. & Visscher, P. M. GCTA: a tool for genome-wide complex trait analysis. *Am. J. Hum. Genet.* **88**, 76–82 (2011).
12. Manichaikul, A. *et al.* Robust relationship inference in genome-wide association studies. *Bioinformatics* **26**, 2867–2873 (2010).
13. UK Biobank secure online repository services. Accessing Bulk Data within UK Biobank. (2019).
14. Wang, K., Li, M. & Hakonarson, H. ANNOVAR: functional annotation of genetic variants from high-throughput sequencing data. *Nucleic Acids Res.* **38**, e164 (2010).
15. Jagadeesh, K. A. *et al.* M-CAP eliminates a majority of variants of uncertain significance in clinical exomes at high sensitivity. *Nat. Genet.* **48**, 1581–1586 (2016).
16. Lek, M. *et al.* Analysis of protein-coding genetic variation in 60,706 humans. *Nature* **536**, 285–291 (2016).
