# Supplementary material for: Calibrated rare variant genetic risk scores for complex disease prediction using large exome sequence repositories
Source: Nat Commun. 2021 Oct 6;12:5852. doi: 10.1038/s41467-021-26114-0 (PMC8494733; doi:10.1038/s41467-021-26114-0)
Supplement: Supplementary file 3 — Description of Additional Supplementary Files [file 41467_2021_26114_MOESM3_ESM.pdf]

Description of Additional Supplementary Files

Title: Supplementary Data 1

Description: Details on overall study design along with pertinent inclusion criteria for cases and controls for each MIGen exome sequencing cohort.

Title: Supplementary Data 2

Description: Characteristics of rare pathogenic alleles observed in LDLR among 5,910 cases from the MIGen consortium.

Title: Supplementary Data 3

Description: Top gene-based discovery associations using expected allele counts that were adjusted with RV-EXCALIBER. Variants included were nonsynonymous SNVs with an M-CAP score >0.025 and all disruptive variants. Odds ratios were ascertained with a Fisher's Exact test and gene-based Pvalues were obtained using RV-EXCALIBER (see Methods). OR indicates odds ratio and CI indicates confidence interval.

Title: Supplementary Data 4

Description: Effect estimates and heterogeneity P-values for 300 RVGRS determined in the UK Biobank and PROMIS exomes. All estimates were calculated using a multivariable logistic regression model that was adjusted for sex and the first 20 principal components of ancestry. P-values for heterogeneity were ascertained with a fixed effect meta-analysis between the β estimate and standard error of the β estimate for the RVGRS constructed using RV-EXCALIBER and TRAPD for a given number of discovery genes.

Title: Supplementary Data 5

Description: Effect estimates of 300 RVGRS (calculated using genebased odds ratios from RV-EXCALIBER or TRAPD) on CAD in the UK Biobank. All odds ratios and P-values were calculated using a multivariable logistic regression model that was adjusted for age, age2 , sex, and the first 20 principal components of ancestry. OR indicates odds ratio and CI indicates confidence interval.
