# Supplementary material for: Calibrated rare variant genetic risk scores for complex disease prediction using large exome sequence repositories
Source: Nat Commun. 2021 Oct 6;12:5852. doi: 10.1038/s41467-021-26114-0 (PMC8494733; doi:10.1038/s41467-021-26114-0)
Supplement: Supplementary file 9 — Reporting Summary [file 41467_2021_26114_MOESM9_ESM.pdf]

nature research

Corresponding author(s): Guillaume Paré

Last updated by author(s): Aug 5, 2021

Reporting Summary

Nature Research wishes to improve the reproducibility of the work that we publish. This form provides structure for consistency and transparency in reporting. For further information on Nature Research policies, see our Editorial Policies and the Editorial Policy Checklist.

Statistics

For all statistical analyses, confirm that the following items are present in the figure legend, table legend, main text, or Methods section.

n/a	Confirmed	
☐	☒	The exact sample size (*n*) for each experimental group/condition, given as a discrete number and unit of measurement
☐	☒	A statement on whether measurements were taken from distinct samples or whether the same sample was measured repeatedly
☐	☒	The statistical test(s) used AND whether they are one- or two-sided<br>*Only common tests should be described solely by name; describe more complex techniques in the Methods section.*
☐	☒	A description of all covariates tested
☐	☒	A description of any assumptions or corrections, such as tests of normality and adjustment for multiple comparisons
☐	☒	A full description of the statistical parameters including central tendency (e.g. means) or other basic estimates (e.g. regression coefficient) AND variation (e.g. standard deviation) or associated estimates of uncertainty (e.g. confidence intervals)
☐	☒	For null hypothesis testing, the test statistic (e.g. *F*, *t*, *r*) with confidence intervals, effect sizes, degrees of freedom and *P* value noted<br>*Give P values as exact values whenever suitable.*
☒	☐	For Bayesian analysis, information on the choice of priors and Markov chain Monte Carlo settings
☒	☐	For hierarchical and complex designs, identification of the appropriate level for tests and full reporting of outcomes
☒	☐	Estimates of effect sizes (e.g. Cohen's *d*, Pearson's *r*), indicating how they were calculated

Our web collection on statistics for biologists contains articles on many of the points above.

Software and code

Policy information about availability of computer code

Data collection	Not applicable.
Data analysis	Handling and extraction of component elements within variant call files (VCFs) was conducted using version 1.9 of bcftools. VCFs were compressed and indexed using version 0.2.6 of tabix. Sample and variant-level quality control of all genetic and phenotypic data was conducted using plink version 1.9. VCF intersection with coverage data from the genome Aggregation Database (gnomAD) and exome enrichment sites used for sequencing Myocardial Infarctino Genetics exome sequencing consortium (MIGen) participants was conducted using version version 2.25.0 of bedtools. Gene-based annotation (refGene), gnomAD frequency annotation, and in-silico pathogenicity annotation for nonsynonymous single nucleotide variants with version 1.0 of the Mendelian Clinically Applicable Pathogenicity Score was conducted using the April 16, 2019 release of ANNOVAR. Functional characterization of discovery genes was conducted using the GO biological processes database, using the March 8, 2013 version release of the GOrilla software (http://cbl-gorilla.cs.technion.ac.il/). All software are open source and are cited in the manuscript. All statistical data analysis was conducted using the R Project for Statistical Computing (version 3.6.0). All code pertaining to RV-EXCALIBER was written boune again shell version 4.2 and in R version 3.6.0, which can be found on the RV-EXCALIBER GitHub repository (https://github.com/GMELab/RV-EXCALIBER).

For manuscripts utilizing custom algorithms or software that are central to the research but not yet described in published literature, software must be made available to editors and reviewers. We strongly encourage code deposition in a community repository (e.g. GitHub). See the Nature Research guidelines for submitting code & software for further information.

Data

Policy information about availability of data

All manuscripts must include a data availability statement. This statement should provide the following information, where applicable:

- Accession codes, unique identifiers, or web links for publicly available datasets
- A list of figures that have associated raw data
- A description of any restrictions on data availability

Individual-level genetic and phenotypic data for the 9 cohorts from MIGen were obtained under authorized access from the database of genotypes and phenotypes (dbGaP) (https://www.ncbi.nlm.nih.gov/gap/). The dbGaP accession numbers and dbGaP hyperlinks for each of the 9 MIGen cohorts are provided in Supplementary Table 1. Restrictions apply to the availability of the MIGen data since the individual-level genetic and phenotypic data are protected due to data privacy laws. The MIGen data is therefore available through controlled access for qualified researchers via an application for authorized access to the National Heart, Lung, and Blood Institute Data Access Committee through the dbGaP authorized access portal (https://dbgap.ncbi.nlm.nih.gov/aa/wga.cgi?page=login). Researchers who would like to obtain the raw data related to MIGen will be presented with a data use certification which requires that the participants will not be re-identified, data be securely stored, and no data will be shared between researchers (who are not identified as study collaborators) or uploaded onto public domains. Any queries pertaining to MIGen data access (including precise conditions of access, contact details for data access requests, and a timeframe for response to data access requests) can be addressed to the National Heart, Lung, and Blood Institute Data Access Committee (nhlbigeneticdata@nhlbi.nih.gov). Genetic data for the 3 GIAB consensus sequences were obtained from the GIAB ftp repository: https://ftp-trace.ncbi.nlm.nih.gov/giab/ftp/release/NA12878_HG001/NISTv3.3/ for NA12878; https://ftp-trace.ncbi.nlm.nih.gov/giab/ftp/release/ChineseTrio/HG005_NA24631_son/NISTv3.3/ for NA24631; https://ftp-trace.ncbi.nlm.nih.gov/giab/ftp/release/AshkenazimTrio/HG002_NA24385_son/NISTv3.3.2/GRCh37/ for NA24385. Data acquisition for the 3 GIAB consensus sequences is further outlined in Supplementary Note 1, section B and can also be found on the Genome In A Bottle (GIAB) Resources webpage (https://jimb.stanford.edu/giab-resources). Individual-level genetic and phenotypic data was also obtained from the UK Biobank (http://www.ukbiobank.ac.uk/), under application #15255, which is further outlined in Supplementary Note 6, section A. Access to the UK Biobank genetic and phenotypic data is also not publicly available and must be obtained via an application (https://www.ukbiobank.ac.uk/register-apply/). Summary-level allele frequency and sequencing coverage information for gnomAD variant sites was obtained from version 2.0.1 of gnomAD exomes (https://gnomad.broadinstitute.org/downloads), which is further described in Supplementary Note 1, section A. Annotated lists of gnomAD variant sites that are stratified by sequencing coverage have been deposited in our public GitHub repository. Lastly, summary statistics containing the beta weights used to generate CVGRS in individuals from the UK Biobank were obtained from the 1000 genomes based CARDIoGRAMplusC4D meta-analysis (http://www.cardiogramplusc4d.org/data-downloads/). Researchers who would like to obtain the raw data related to this study will be presented with a data use certification which requires that the participants will not be re-identified, data be securely stored, and no data will be shared between researchers (who are not identified as study collaborators) or uploaded onto public domains. Source data are provided with this paper.

Field-specific reporting

Please select the one below that is the best fit for your research. If you are not sure, read the appropriate sections before making your selection.

☒ Life sciences ☐ Behavioural & social sciences ☐ Ecological, evolutionary & environmental sciences

For a reference copy of the document with all sections, see nature.com/documents/nr-reporting-summary-flat.pdf

Life sciences study design

All studies must disclose on these points even when the disclosure is negative.

Sample size	Sample sizes were determined from the available data (i.e. from MIGen and the UK Biobank).
Data exclusions	Extensive quality control was conducted to remove samples and variants of low quality for both MIGen and the UK Biobank as described in Supplementary Notes 3-5. Regions of the genome were also confined to coding sites that intersected exome enrichment sites of MIGen or the UK Biobank and high coverage sites of gnomAD (see Supplementary Note 8, section B). For GIAB samples, regions of the genome were confined to coding sites that intersected the NIST high quality regions and gnomAD high coverage regions (see Supplementary Note 8, section B).
Replication	The association of the rare variant genetic risk score on coronary artery disease (CAD) that was established in the UK BioBank (n=3,843 CAD cases and n=42,007 CAD controls) was successfully replicated in the Pakistan Risk of Myocardial Infarction Study (PROMIS) which contained 2,946 myocardial infarction (MI) cases and 3,708 MI controls.
Randomization	Not applicable. Our study is an observational study design and is not a randomized trial.
Blinding	Not applicable. Our study did not involve any types of interventions that would otherwise require blinding.

Reporting for specific materials, systems and methods

We require information from authors about some types of materials, experimental systems and methods used in many studies. Here, indicate whether each material, system or method listed is relevant to your study. If you are not sure if a list item applies to your research, read the appropriate section before selecting a response.

Materials & experimental systems

n/a	Involved in the study
☒	☐ Antibodies
☒	☐ Eukaryotic cell lines
☒	☐ Palaeontology and archaeology
☒	☐ Animals and other organisms
☐	☒ Human research participants
☒	☐ Clinical data
☒	☐ Dual use research of concern

Methods

n/a	Involved in the study
☒	☐ ChIP-seq
☒	☐ Flow cytometry
☒	☐ MRI-based neuroimaging

Human research participants

Policy information about studies involving human research participants

Population characteristics	Post quality control population characteristics for the cohorts encompassing MIGen (n=11,992) are provided in Supplementary Table 1, including proportion of individuals who were male/female and those passing sample quality control metrics as outlined in supplementary section 6. All post quality control participants in MIGen were of European ancestry, with exception to participants from the PROMIS cohort, which were of South Asian ancestry. Phenotype information for MIGen was obtained from dbGaP, but there was no information on the age of study participants. Genotype information for MIGen participants was also downloaded from dbGaP in VCF format, and was generated from whole exome sequencing data using Illumina sequencing technology (summarized in supplementary table 1). UK Biobank participants passing quality control (n=45,850) had an age range of 40-70 where 54% were female and 46% were male. Genotype information for UKB participants was obtained in gVCF format from whole exome sequencing data, which was generated using the functional equivalent pipeline. All MIGen and UK Biobank participants used in this study were of European ancestry.
Recruitment	Study participants from MIGen were recruited based on pre-defined inclusion and exclusion criteria (per-cohort) for cases and controls as outlined in Supplementary Data 1. UK Biobank participants were recruited in an unselected manner as part of a longitudinal study design. No local recruitment of cases was conducted.
Ethics oversight	For the MIGen, all data handling for individual-level phenotype and genotype information along with the proposed analyses were approved by the Hamilton Integrated Research Ethics Board and approval for data downloads was subsequently granted by the National Heart, Lung, and Blood Institute Date Authorization Committee (NHLBI DAC). For the UK Biobank phenotype and genotype data were accessed in March 2019 under application #15255 and contained individual-level genotype and phenotype data for individuals consenting to genetic analysis for health research.

Note that full information on the approval of the study protocol must also be provided in the manuscript.
